# Pulmonary Function and Sleep-Related Disorders During Cervical Administration of Intrathecal Baclofen in Adults With Spinal Cord Injury (Cervit-B): Protocol for a Multiphase Single-Arm Intervention Trial

**DOI:** 10.2196/70362

**Published:** 2025-08-14

**Authors:** Ellen M Maas, Kuan H Kho, Johan S Rietman, Marjolein G J Brusse-Keizer

**Affiliations:** 1 Roessingh, Centrum voor Revalidatie Enschede The Netherlands; 2 Department of Neurosurgery Medisch Spectrum Twente Enschede The Netherlands; 3 Department of Clinical Neurophysiology, Technical Medical Center, University of Twente Enschede The Netherlands; 4 Faculty of Engineering Technology, Technical Medical Center, University of Twente Enschede The Netherlands; 5 Medical School Twente, Medisch Spectrum Twente Enschede The Netherlands; 6 Health Technology & Services Research, Technical Medical Centre, University of Twente Enschede The Netherlands

**Keywords:** spinal cord injury, spasticity, intrathecal baclofen, upper extremity, pulmonary function, sleep-related disorders

## Abstract

**Background:**

The effect and safety of intrathecal baclofen (ITB) on generalized spasticity of the lower extremity have been well described in numerous studies, whereas the safety and effect on spasticity of the upper extremity during cervical administration seems to be less certain.

**Objective:**

We aim to establish the safety of cervical administration of ITB on pulmonary function and sleep-related disorders in adults with a cervical spinal cord injury with functional hindering spasticity. In addition, we aim to explore the effect of cervical ITB on the reduction of spasticity; participant satisfaction; and improvement at the level of function, activities, and participation.

**Methods:**

The study is a multiphase single-arm intervention study. Before the start of the study, participants are screened for the presence of sleep-related disorders, and pulmonary function is assessed using spirometry and a capillary blood gas sample. Participants are allowed to start the first phase of the study if there are no signs of sleep-related disorders or if these are adequately treated. The P_c_CO_2_ (partial pressure of CO_2_ in capillary blood sample) should be between 4.5 and 6.5 kPa. In the first phase of the study, an extracorporeal pump will be used to investigate whether cervical administration of ITB leads to a reduction of spasticity without an adverse effect on pulmonary function and sleep-related disorders. In case of a safe (no adverse effects) and positive (reduction of spasticity) result of the trial, a baclofen pump will be implanted in the second phase of the study. After implantation, the dosage of ITB will be slowly increased, and eventually, the use of oral spasmolytics will be phased out. The dosage will be increased during the monitoring of pulmonary function and sleep-related disorders. In the second phase of the study, a pulse oximetry will be performed, and pulmonary function; spasticity; satisfaction; and the level of function, activities, and participation will be assessed at 3, 6, and 12 months after definitive implantation of the baclofen pump.

**Results:**

The first participant was recruited on May 23, 2023. Data collection and analyses are expected to be completed in 2027.

**Conclusions:**

Currently, little is known about the safety and effectiveness of cervical administration of ITB for the treatment of upper extremity spasticity. We aim to establish the safety and explore the efficacy of this procedure. This paper describes the protocol of this study.

**International Registered Report Identifier (IRRID):**

DERR1-10.2196/70362

## Introduction

### Background

Cervical spinal cord injury (SCI) is often accompanied by spasticity of the upper extremity (UE), leading to limited function of the UE, which may negatively influence the quality of life [1]. Therefore, effective treatment of spasticity is of great importance. Neuromuscular blocks or surgery are treatments for focal spasticity. Neuromuscular blocks might be insufficient for generalized spasticity, given the maximum tolerated dose and potential complications [2]. Baclofen therapy is well established for the treatment of generalized spasticity [2]. Baclofen is a strong and selective gamma-aminobutyric acid-B receptor agonist, leading to presynaptic inhibition of reflex activity. Dosing the baclofen directly into the spinal fluid results in a higher drug concentration at the site of action, with fewer systemic side effects compared to oral baclofen. The effect of intrathecal baclofen (ITB) on generalized spasticity of the lower extremity (LE) has been well described in numerous studies, whereas the effect on spasticity of the UE seems to be less certain [2,3]. This difference in effectiveness might be related to the placement of the catheter tip intrathecally and the baclofen gradient in the cerebrospinal fluid [4,5]. In current settings, the catheter tip is usually placed at the thoracolumbar level.

It has been shown that the concentration of baclofen is highest around the tip of the catheter and the concentration rapidly decreases in caudal and rostral direction [4,5]. Furthermore, the rate of decrease depends on the method of infusion (bolus or continuous). There is a faster decrease in concentration in continuous infusion [5].

As the catheter position affects the concentration gradient, the concentration of ITB at the cervical level will be higher if the catheter tip is at the cervical level than if the catheter tip is at the thoracolumbar level. To properly treat spasticity of the UE, the tip of the catheter should be positioned cervically. A cervical catheter tip will probably, in addition to the effect on spasticity of the UE, also have an effect on the spasticity of the respiratory muscles.

Reducing the spasticity of respiratory muscles could improve respiration and pulmonary function [6-8]. However, higher concentrations of ITB at the cervical level may lead to a deterioration of pulmonary function and an increased risk or increased severity of sleep apnea syndrome (SAS) due to respiratory muscle weakness and a central dampening effect [3].

A systematic review by Jacobs et al [9] suggests that cervically administered ITB seems to improve UE spasticity and function, without causing more complications than thoracolumbar ITB. However, the main drug-related complications have not been thoroughly investigated, preventing definitive conclusions [9]. This conclusion is confirmed in a recent study by Mossner et al [10]. Therefore, we propose investigating the safety and exploring the efficacy of this procedure.

### Objective

We aimed to extensively describe the design and methodology of the Cervit-B study, in which we will investigate the safety of cervical administration of ITB in adults with SCI. In addition, we will explore the effect of cervical ITB on spasticity; the level of function, activities, and participation; and participant satisfaction. We hypothesize that cervical administration of ITB is a safe procedure without adverse effects on pulmonary function, as measured by spirometry and capillary blood gas, and on sleep-related disorders in the sense of SAS. In addition, we hypothesize that it improves the efficacy of spasticity management, enhances functional abilities and activities, promotes greater social participation, and increases participant satisfaction.

## Methods

### Study Design

This multiphase single-arm intervention study will contain 2 phases. We will first start with screening and recruitment to determine if there is an indication for, and if it is safe to start treatment with ITB. Phase 1 will be the safety phase, in which an extracorporeal pump will be used to investigate whether cervically administered ITB leads to reduction of spasticity without adverse effect on pulmonary function and sleep-related disorders. In case of a safe and positive test, we will continue to the next phase (phase 2) in which a baclofen pump is implanted. After implantation, the dosage of ITB will be slowly increased to the optimal setting, and if the participant uses oral spasmolytics, these are phased out. The optimal setting varies in accordance with patient conditions. In this phase of the study, we will focus, in addition to pulmonary function and sleep-related disorders, on the reduction of spasticity; improvement of participants’ level of function, activities, and participation; and participant satisfaction according to the International Classification of Functioning [11].

All participants will be assessed once before the start of phase 1 (T0A1), at the start of phase 1 (T0A2), and daily during phase 1 (T0B). In the case of a safe and positive test, participants will be assessed at the end of phase 1 (T1). During phase 2, they will be assessed at 3, 6, and 12 months (T2, T3, and T4) after baclofen pump implantation. We chose to start the assessment in phase 2 only after 3 months following the implantation because in the first 3 months, the pump settings will still be regularly adjusted to optimize the daily dosage of ITB.

### Setting

The study settings of phases 1 and 2 will be at the Medisch Spectrum Twente (MST) hospital and Roessingh Center for Rehabilitation (RCR), respectively. Both the centers are in Enschede, the Netherlands.

### Study Coordination

RCR is the coordinator of the study and is responsible for the study design, management, data collection, and data analysis.

A data safety monitoring board is involved throughout the study. Once the first 6 participants have completed phase 1, the board will assess the safety and related continuation of the study. Monitored visits by an independent study monitor are planned every 6 months. This is to ensure the continued protection of participants’ rights and well-being, assure protocol adherence, and verify that data integrity during the study complies with good clinical practice [12]. All protocol deviations will be filed. In this study protocol, the SPIRIT (Standard Protocol Items: Recommendations for Interventional Trials) reporting guidelines are used [13].

### Ethical Considerations

The study will be conducted according to the principles of the Declaration of Helsinki, (October 2013 version), and in accordance with the Medical Research Involving Human Subjects Act (WMO). Approval of the study has been obtained from the Medical Ethics Committee Oost Nederland (2021-7364) and the Central Committee on Research Involving Human Subjects. The trial is registered in the EudraCT database (2021-004994-30) and is transitioned to the Clinical Trials Information System (2023-508143-51-01). All administrative and protocol-related amendments will be submitted for approval to the Medical Ethical Committee. After receiving their approval, these modifications will be reported via email to the participating centers and the external monitor. The sponsor has both patient and liability insurance, which is in accordance with article 7 of the WMO.

After the nature and possible consequences of the study are explained, informed consent will be obtained from all participants. Participants will be informed that they always have the ability to opt out. Apart from travel expenses, there is no compensation for the participants.

Before the start of the study, a data management plan that covers all aspects of handling data gathered during and after completion of the study was devised.

All documents related to the study and all participants’ data will be collected in the investigators’ site files. These files, containing personal and contact information, as well as the screening information from potential candidates, will be safeguarded in RCR. All relevant data will be copied from the case report form into a web-based clinical database for case report data (Castor, Castor EDC) by the primary investigator using unique and anonymous participant codes. If necessary, this study identification number can be linked to the participant by the primary investigator only. The primary investigator, the research assistant in RCR, the research nurse in MST, and the monitor will have access to the full dataset.

All coded data will be stored for 10 years within the clinical database.

### Participants

Adults with SCI eligible for the study must have generalized or multifocal spasticity in UE and eventually also in the LE. Their spasticity must be refractory to oral drugs or local treatment [14,15].

In the Netherlands, there are 8 rehabilitation centers with a specialized spinal cord unit. These 8 centers work closely together on patient-related content in the Dutch-Flemish Spinal Cord Society. According to guidelines in the Netherlands, all persons with SCI in the chronic phase are seen by a rehabilitation physician [16-18]. The rehabilitation physician involved in the chronic phase is familiar with the Cervit-B study. In case of generalized therapy-resistant spasticity of the UE and eventually also the LE, patients are referred to RCR by their rehabilitation physician. In RCR, they are seen by the principal investigator (EMM) for assessment to check the indication for cervical administration of ITB. Spasticity is assessed using the Perceived Resistance to Passive Movement (PRPM) and the Modified Ashworth Scale (MAS) [19,20].

When there is an indication for cervical administration of ITB, participants will be informed about the study. When participants are interested, an information letter is given to them. After 1 week, the principal investigator (EMM) will contact the participants to determine their interest in participating in the study and to answer possible questions. If interested in participating, a physical appointment with the principal investigator (EMM) is scheduled to obtain informed consent and to complete the screening procedure to check if it is safe to start phase 1. If it is safe, participants will be seen by the skilled functional neurosurgeon at the outpatient clinic in MST before the start of phase 1. The neurosurgeon will check for operative exclusion criteria and will answer questions. The same neurosurgeon will be involved in the study during all surgical procedures and admission at MST.

The inclusion and exclusion criteria for the Cervit-B study are described in Textbox 1 [21-23].

**Inclusion and exclusion criteria.**

**Inclusion criteria**

- Aged >18 years
- Spinal cord injury (SCI) with a neurological level at or above Th1
- >1 year after the onset of SCI
- An American Spinal Injury Association Impairment Scale of A to D, according to the International Standards for Neurological Classification of Spinal Cord Injury [21]
- Generalized or multifocal spasticity in UE, and eventually also in LE, refractory to oral drugs or local treatment
- Providing written informed consent

**Exclusion criteria**

- Pregnancy
- Women of childbearing potential
- Women who are currently breastfeeding
- Baclofen allergy
- Use of oral anticoagulants
- Individuals with active depression, schizophrenia, or bipolar disorders who are still under treatment by a psychiatrist, as exacerbations of these conditions during the use of baclofen have been observed [22]
- Excessive alcohol use according to the Diagnostic and Statistical Manual of Mental Disorders, Fifth Edition [23], because interactions with alcohol are unpredictable [22]
- Progressive disease as the cause of SCI
- Concomitant presence of a terminal disease, such as cancer
- Muscle or nerve blocks carried out in the last 6 months
- Dependent on invasive or noninvasive ventilation other than a Continuous Positive Airway Pressure device
- P_c_CO_2_ (partial pressure of CO_2_ in capillary blood sample)>6.5 kPa or <4.5 kPa
- Not adequately treated sleep apnea syndrome with an Apnea-Hypopnea Index (AHI) >5. The AHI represents the average number of apneas and hypopneas each hour during sleep.

### Sample Size

**Figure 1:**
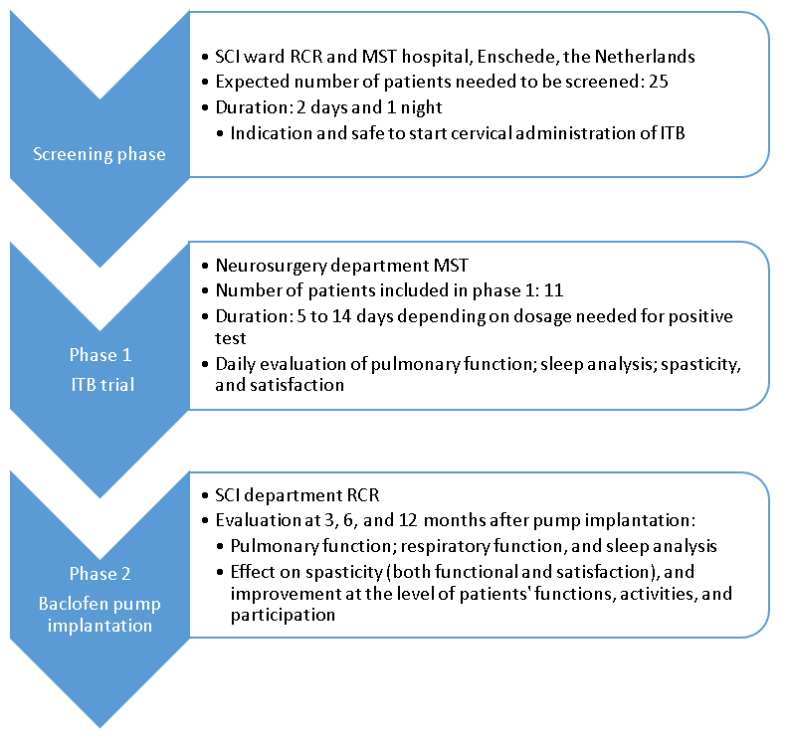
Flowchart of the study phases, including settings, sample size, and parameters to be assessed. ITB: intrathecal baclofen; MST: Medisch Spectrum Twente; RCR: Roessingh Center for Rehabilitation; SCI: spinal cord injury.

No sample size calculation was performed for this pilot safety study. On the basis of a previous comparable study by Bensmail et al [3] with 11 participants, we decided to include the same number of participants. We expect that we need to screen and recruit 25 participants to retain 11 participants at the start of the phase 1 because of the safety criteria for inclusion (Figure 1).

### Study Procedures

#### Screening and Recruitment

Before participants are included in the study, they will be assessed by an experienced SCI rehabilitation physician to check the indication for treatment of their spasticity by ITB. If there is an indication, participants will be screened for the presence of SAS by the use of OSAsense (OSAsense BV) [24]. The OSAsense consists of a validated online questionnaire and a nightly saturation and pulse rate measurement (pulse oximetry). The OSAsense algorithm, based on the questionnaire and pulse oximetry, is able to detect SAS with a sensitivity of 100%, a specificity of 35%, a negative predictive value of 100%, and a positive predictive value of 79.4% [25]. If, according to the algorithm, there is any suspicion of SAS, polysomnography will be performed, and the patient will be set up for SAS treatment if necessary. In addition, a morning capillary blood gas sample will be taken to determine the P_c_CO_2_. We chose to take a capillary blood gas sample because there is a strong correlation between samples collected from the fingertip capillaries and the arterial blood samples in relation to the analysis of blood [26].

These measurements determine the safety for participants to start the first phase of the study. The start of the first phase of the study will be determined as safe if the participant is stable enough to undergo surgery (defined by neurosurgeon), capillary blood gas shows a P_c_CO_2_ between 4.5 and 6.5 kPa, and there are no symptoms of SAS or, in the case of SAS, the participant is adequately set to SAS treatment with an Apnea-Hypopnea Index (AHI) <5. These cut-off values have been chosen according to the applicable guidelines in the Netherlands [27].

#### ITB Trial (Phase 1)

Before definitive treatment with ITB, individuals will undergo an ITB trial by continuous infusion using an extracorporeal pump. We opted for this type of trial treatment based on a study by Bensmail et al [3], which demonstrated that a bolus infusion mode gave relatively more increase in the respiratory disturbance index and of central and mixed sleep apneas than the continuous infusion mode.

During phase 1, participants will be admitted to MST in Enschede. A hybrid infusion system consisting of a spinal catheter (Medtronic Ascenda 8781; Conformité Européenne [CE] mark for intrathecal use) and an implantable infusion port (Cellsite spinal; CE mark for intrathecal use) will be implanted under deep sedation. The participant lies in a lateral decubitus position. After marking the incision locations in the midlumbar region and the paraumbilical area, the incision areas will be infiltrated with local anesthesia (bupivacaine). After sterile draping, the lumbar fascia is exposed through a 2- to 3-cm incision, and a 16 T-gauge introducer needle will be placed to access the intrathecal space. Then, the spinal catheter will be placed under fluoroscopic guidance to the cervical level just below the level of injury. Once positioned, an anchor will be placed over the catheter and sutured to the deep fascia of the musculus erector spinae. The catheter will then be tunneled subcutaneously from the midlumbar region to the paraumbilical area and will be connected to the subcutaneous infusion port. Before wound closure, the port will be accessed to document the cerebrospinal fluid flow through the complete hybrid system. Thereafter, an extracorporeal pump (CADD solis infusion pump, CE mark for intrathecal use or application) will be connected percutaneously to the subcutaneous infusion port.

For safety reasons, we chose to start with the lowest possible dose given the concentration of baclofen and the infusion rate of the extracorporeal pump used. The starting dose of the ITB will be 1 µg per hour and is titrated in the following days based on both objective and subjective (participant’s experience) change in spasticity. In the literature, no maximum dose of ITB during a trial with an extracorporeal pump is reported. We set the maximum dose at 200 µg per day, based on our extensive previous experiences with the use of an extracorporeal pump in ITB trials.

Daily capillary blood gas samples will be taken, and continuous pulse oximetry during sleep, by use of the OSAsense, will be recorded. If the participant is set on a continuous positive airway pressure (CPAP) device, the CPAP device is read out. The trial will be determined as unsafe and will be stopped if the P_c_CO_2_ is >6.5 kPa for 2 days in a row, according to hypoventilation rules in adults as mentioned by Berry et al [28]. The trial will also be stopped when participants without SAS, during the trial, will develop complaints matching SAS and the Oxygen Desaturation Index (ODI) is between 5 and 15 [27]. The ODI represents the number of desaturations per hour.

A third reason to stop the trial is when participants during the trial have no complaints matching SAS, but the ODI is >15 [27]. If the participant uses a CPAP device, the setting of the device may be adjusted to stay within the safety margin. This safety margin is set at an AHI <5 [24]. This adjustment will be registered. A fourth reason to stop the study is when, after adjustments of the CPAP device, it is not possible to stay within this safety margin [24]. A fifth reason to stop the study is if there is no effect on spasticity at the maximum dose of ITB (200 µg per day).

If the participant experiences a positive effect of the ITB on their spasticity, with a score between 1 and 3 on the Patient Global Impression of Change (PGIC) [29] without deterioration on functionality (PGIC 5-7) and there is not a clinically relevant deterioration of pulmonary function or SAS, it is considered a safe and positive ITB trial with an indication for intracorporeal pump implantation (phase 2) and treatment with ITB at cervical level. We opted for these cut-off values for assessment of reduction of spasticity according to the algorithm established by the European working group for spasticity in adults [30].

**Figure 2:**
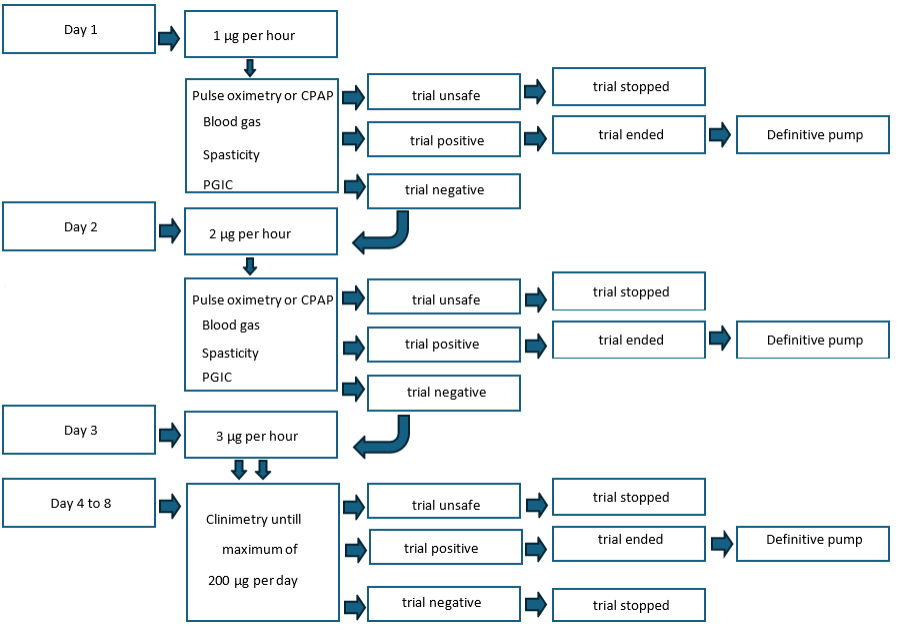
Overview of phase 1. CPAP: continuous positive airway pressure; PGIC: Patient Global Impression of Change.

If, during the trial, no effect is observed on spasticity or there is a clinically relevant deterioration of pulmonary function or SAS, the trial will be discontinued. This is considered a negative test in which there is no indication for treatment of spasticity with ITB. The catheter and the subcutaneous infusion port will then be removed (Figure 2)*.*

#### Baclofen Pump Implantation (Phase 2)

In the case of a positive ITB trial phase, the infusion port will be replaced by a definitive pump system. In this procedure, the participant is in a supine position and under deep sedation or local anesthesia. The infusion port is removed from the catheter, and the catheter is connected to a programmable implantable pump (Medtronic SynchroMed II; CE mark) for intrathecal drug delivery.

One day after the pump implantation at the MST, participants will be transferred to RCR. Participants will remain in the rehabilitation center for at least 2 weeks. During admission, the dosage of ITB will be slowly increased, and the oral spasmolytics will be phased out. Spasticity is measured on a daily basis, and for safety reasons, once a week and at discharge, a capillary blood gas sample is taken and a screening with the OSAsense is performed, or CPAP data are checked. The participant will be discharged home when the oral spasmolytics are phased out, the patient is satisfied with the current level of spasticity (PGIC between 1 and 3), and the AHI or ODI and P_c_CO_2_ are within the safety margins, as indicated previously. The participant will be seen for follow-up at the rehabilitation center at 3, 6, and 12 months after implantation. To promote follow-up, the participants will be called once before their appointment by the primary investigator. In case a participant stops or deviates from the protocol, the reason for this will be recorded.

### Outcomes, Characteristics, and Other Variables

#### Overview

Table 1 shows an overview of the primary and secondary outcome measurements, descriptive variables, and timing of the data collection. At both centers, everyone involved in the measurements is trained in measuring and collecting study data in a uniform and reproducible manner. A training and delegation log will be maintained by the principal investigator (EMM).

**Table 1:** Overview of outcome measures and timing.

Construct			Timing																	Measures
			Screening		First phase (test phase)							Second phase (after implantation)								
			T0A1^a^		T0A2^b^: before starting the trial		T0B^c^: during the trial		T1^d^: end of the trial		T2^e^			T3^f^		T4^g^				
**Primary outcomes**																				
	Respiratory function	✓		✓		✓		✓		✓			✓		✓		Capillary blood gas			
	Sleep disorder	✓				✓				✓			✓		✓		Pulse oximetry^h^			
	Pulmonary function	✓		✓				✓		✓			✓		✓		Spirometry^i^			
**Secondary outcomes**																				
	Patient satisfaction					✓		✓		✓			✓		✓		PGIC^j^			
	Spasticity	✓		✓		✓		✓		✓			✓		✓		PRPM^k^ and MAS^l^			
	Pinch and grip strength	✓								✓			✓		✓		e-link			
	Muscle power	✓								✓			✓		✓		MRC^m^ scale			
	Passive ROM^n^	✓								✓			✓		✓		Degrees			
	Walking ability (if possible)	✓								✓			✓		✓		Timed Up and Go Test and 10-Meter Walking Test			
	Activity and participation	✓								✓			✓		✓		COPM^o^, GRT^p^, and QIF-sf^q^			
**Participants and disease characteristics**																				
	Sex	✓																		
	Race	✓																		
	Age	✓																		
	Level and completeness of the SCI^r^	✓															International Standards for Neurological Classification of Spinal Cord Injury			
	Etiology of SCI	✓																		
	Time since injury	✓																		
	Other treatments against spasticity	✓																		
	Medication	✓		✓		✓		✓		✓			✓		✓					
	Comorbidity	✓		✓		✓		✓		✓			✓		✓					
	Vital signs	✓		✓		✓		✓												
	Complications and side effects			✓		✓		✓		✓			✓		✓					

^a^T0A1: preassessment screening.

^b^T0A2: start of the extracorporeal pump.

^c^T0B: period during the trial phase.

^d^T1: moment appropriate titration in the trial phase.

^e^T2: 3 months after implantation.

^f^T3: 6 months after implantation.

^g^T4: 1 year after implantation.

^h^If there is any suspicion of sleep apnea syndrome based on the results of the pulse oximetry, additional polysomnography will be performed during the screening and recruitment.

^i^If participants during the trial phase experience dyspnea, spirometry will be performed.

^j^PGIC: Patient Global Impression of Change.

^k^PRPM: Perceived Resistance to Passive Movement.

^l^MAS: Modified Ashworth Scale.

^m^MRC: Medical Research Council.

^n^ROM: range of motion.

^o^COPM: The Canadian Occupational Performance Measure.

^p^GRT: Grasp and Release Test.

^q^QIF-sf: Quadriplegia Index of Function—Short Form.

^r^SCI: spinal cord injury.

#### Primary Outcome Measures

The primary objective of the study is to establish whether cervical administration of ITB is a safe treatment for spasticity without deterioration of pulmonary function and possible increased risk or increased severity of SAS. Safety will be determined by use of a capillary blood gas sample and an OSAsense or CPAP screening before and during the first phase of the study. We will use P_c_CO_2_ and the AHI or ODI as safety markers.

#### Secondary Outcome Measures

The secondary objective of the study is to explore the effect of cervical administration of ITB on the reduction of spasticity; intermediate and long-term effects on pulmonary function and SAS; and improvement in the level of participants’ function, activities, and participation [11]. Participant satisfaction will also be considered.

All secondary outcome measures are carried out in RCR at screening and at 3, 6, and 12 months after implantation of the baclofen pump by an experienced and trained physiotherapist and occupational therapist. Spasticity and satisfaction will also be measured during phase 1.

Spasticity is measured by the MAS [19] and PRPM [20]. Spasticity will be measured in the UE and LE. The PRPM is an ordinal scale that measures and describes perceived resistance against passive motion. The scale ranges from 0 (no increase in resistance) to 4 (limb is rigid in flexion or extension). During testing, the patient is in a relaxed position (the position of the patient is documented). The patient is instructed not to assist or oppose the movement. First, the passive range of motion of the joint is tested by slow flexion or extension. Subsequently, perceived resistance is assessed within the entire range with a faster movement, covering the whole range within 1 second. The movement can be repeated a maximum of 2 times (the lowest score is noted).

The MAS measures the increase in muscle tone and assigns a grade of spasticity from a 0 to 4 ordinal scale. A score of 0 signifies normal muscle tone, a score of 1 signifies a slight increase in muscle tone with a slight catch when the limb is moved in flexion and extension, a score of 1^+^ signifies a slight increase in muscle tone with minimal resistance at the end of movement, a score of 2 signifies a more marked increase in muscle tone through the entire movement but the limb is still easy to move, a score of 3 indicates a considerable increase in muscle tone and that passive movement is difficult, and a score of 4 indicates that the limb is rigid in flexion or extension. The position of the patient and the procedure is the same as in the PRPM.

Participant satisfaction will be measured by PGIC [29], which is a 7-point scale ranging from 1 (very much improved) to 7 (very much worse). Using the scale, the participant can rate if the ITB has changed the level and the hindrance of the spasticity according to the situation before cervical administration of ITB.

Muscle strength of the core muscles of the UE and LE will be measured by the Medical Research Council (MRC) scale [31]. The core muscles of the upper extremity are the elbow flexors, the wrist dorsal flexors, the elbow extensors, the deep flexor of digit 3, and the ulnar abductor of digit 5. The core muscles of the LE are the hip flexors, the knee extensors, the ankle dorsal flexors, the extensors of digit 1, and the ankle plantar flexors. The MRC scale is an ordinal scale ranging from 0 to 5. With a score of 0, no muscle activity is seen, a score of 1 indicates muscle contraction is felt or observed but no full range of motion, a score of 2 indicates that there is a full range of motion with gravity eliminated, a score of 3 suggests a full range of motion against gravity, a score of 4 indicates that there is muscle activity against some resistance, and a score of 5 suggests a normal force. During testing, the patient is in supine position and tight or restrictive clothing is removed so the examiner can visualize the muscles being tested and observe for muscle twitch. Muscles are first tested with gravity eliminated so that muscle contraction is perpendicular to gravity. If the participant is unable to engage the muscle with gravity eliminated, the examiner places a hand on the muscle and asks the participant to contract his or her muscles again. This allows the examiner to feel for a muscle twitch, even if a twitch is not visible. This observation would differentiate a score of 0 from a score of 1. When the participant demonstrates the full range of motion with gravity eliminated, the test should be repeated against gravity for the full range of motion. If this is successful, the examiner should challenge the participant by the addition of a small degree of resistance, and then by the maximum resistance to differentiate between a score of 3, 4, or 5.

For the lateral- and cylindrical grip strength, the E-Link Hand Grip Dynamometer and Pinchmeter (Biometrics Ltd) will be used [31]. For the measurement, the participant sits on a chair or in his or her wheelchair at a table with the arm resting on the table with the elbow at a 90° angle. The participant should squeeze as hard as possible and hold this for about 2 seconds. Each hand is measured 3 times and a mean score is calculated.

For the measurement of passive range of motion, a goniometer is used. The participant is in a supine position. This measurement is performed by two physiotherapists, with one therapist moving the joint and the other using the goniometer to record the joint’s mobility in degrees. Notation is done via the neutral 0 method [32].

Walking ability and the ability to safely stand up from a chair will be measured by the Timed Up and Go test [32] and the 10-Meter Walking Test [33].

The Timed Up and Go test measures the time it takes the participant to get up from a chair or a wheelchair, walk 3 meters comfortably (most energetically efficient), turn around, walk back, and sit down. At the start, the participant sits in the chair or the wheelchair with hands on the upper legs. When standing up and sitting down, the armrests may be used. Time is stopped when the participant sits again with their hands on their legs. The participants may use their own walking aid or orthosis, but no physical help should be provided.

The 10-Meter Walking Test measures the speed of comfortable walking and the maximum walking speed over a distance of 10 meters. If necessary, it is permitted to use a walking aid or orthoses, but the patient must be able to walk without physical help.

For the assessment of the level of activities and participation, the Canadian Occupational Performance Measure [34], the Grasp and Release Test [35], and the Quadriplegia Index of Function—Short Form (QIF-sf) [36] will be used.

The Canadian Occupational Performance Measure [34] is a semistructured interview designed to identify and prioritize everyday issues that restrict individuals’ participation in everyday living. An occupational therapist determines, together with the participant, which 5 activities of daily living he or she would like to be able to perform better and be more independent. These activities are scored on a 10-point scale for both performance and satisfaction.

The Grasp and Release Test [35] measures unilateral hand performance during the manipulation of 6 objects of different sizes and weights—3 objects with lateral pinch (peg, weight, and fork) and 3 objects with palmar grasp (block, can, videotape). The participant sits on a chair or in a wheelchair in front of a table. The position of the participant in relation to the table and the test is exactly documented so that during repeated measurements, this same position can be maintained. Participants are asked to grasp, lift, and release the objects as many times as possible within 30 seconds. Each object is repeated 3 times and a mean score is calculated.

The QIF-sf [36] is a clinician-administered interview, developed to provide a functional assessment for documenting small but clinically significant gains made by SCI quadriplegics. On the basis of the Quadriplegia Index of Function originally developed in 1980, the QIF-sf assesses only 6 activities of daily living instead of 37 in the original version. Items include washed or dry hair, turning supine to side in bed, LE dressing, opening a carton or jar, transferring from bed to wheelchair, and locking the wheelchair. The QIF-sf is scored on a 4-point Likert scale ranging from totally dependent (score of 0) to independent (score of 4).

Pulmonary function [37] will be mapped by spirometry. In RCR, spirometry is performed by a handheld spirometer. Before starting the test, patient’s age, sex, height, and origin are determined for assessment and interpretation of results. The participant is sitting in an upright position, and a nose clip is applied. Thereafter, a mouthpiece is placed into the mouth. The participant is instructed on how to perform the test. The mouthpiece must be surrounded with lips and teeth; the blowhole may not be closed with the tongue. Then, the participant performs a maximum inspiration followed by a fast, forceful, smooth, and full expiration of at least 6 seconds, and finally a rapid, forceful, and complete inspiration.

For the assessment of the spirometry, specific attention is paid to forced vital capacity, forced expiratory volume in 1 second, and peak expiratory flow because of the importance of these parameters on pulmonary function in people with cervical SCI [37]. The forced vital capacity represents the lung capacity when forced to inhale and exhale. The forced expiratory volume in 1 second value shows how much air of the total lung capacity can be exhaled in the first second. The peak expiratory flow concerns the maximum airflow rate during the test.

P_c_CO_2_ is determined by a morning capillary blood gas sample from the fingertip [24]. Sleep analysis is done by pulse oximetry using the OSAsense. The OSAsense consists of a validated online questionnaire and a nightly saturation and pulse rate measurement by a plaster around a finger [24,25]. In case a participant has a CPAP device, the AHI is determined by analyzing the data recorded by the device.

#### Baseline Characteristics and Other Factors

Participants characteristics, such as age; sex; race; neurological level of SCI according to the International Standards for Neurological Classification of Spinal Cord Injury [21]; etiology of SCI, time since SCI; vital signs (temperature, blood pressure, pulse rate and saturation); other treatments for spasticity before inclusion; and comorbidity will be registered at baseline. Medication will be registered at baseline and during assessments at phases 1 and 2.

Other factors that will be registered during the study are possible complications and side effects of ITB, such as temporary liquor loss, infection, epilepsy [37,38], and drowsiness. Serious adverse events and Suspected Unexpected Serious Adverse Reactions will be registered according to applicable guidelines.

### Statistical Analysis

Because of the small sample size, it is expected that the continuous variables will not be parametrically distributed; therefore, they will be presented as median with IQR. Furthermore, due to the small sample size, pre- and postassessments during the first phase or the ITB trial phase will be compared with descriptive statistics and differences will not be formally tested. In addition, the measurements in the second phase after definitive implantation of the baclofen pump (3, 6, and 12 months after implantation) will be compared with descriptive statistics. Changes over time in the outcome measures within patients will be visually explored.

For categorical variables, we will present numbers and corresponding percentages. To compare pre- and postassessment during the first phase or the ITB trial, cross tabs will be made. Our primary analysis population is the intention-to-treat analysis. Considering the study design, we expect a few missing data. In case of missing data, we will use generalized estimation equations analysis (<20% missing data) or multiple imputations (>20% missing data).

## Results

The study has started in January 2021. Funding was completed in March 2022. The first participant was enrolled in May 2023. As of June 2025, we have enrolled 6 patients in phase 1 and 3 patients in phase 2. No data analysis has been done yet. We expect data collection to be completed and published in 2028.

## Discussion

### Anticipated Findings

A literature review [9] on cervical ITB suggests improvement of UE spasticity and function, without causing more complications than thoracolumbar ITB. However, the data prevent definite conclusions. In 2024, a retrospective case series by Mossner et al [10] was performed in children with hypertonia, highlighting the safety and efficacy of using cervical ITB. They concluded that the cervical catheter tip location for ITB was safe, was effective in controlling tone, and should be considered for the treatment of hypertonia. Larger studies with longer follow-up were suggested to further determine upper-limit dosing safety, along with long-term functional benefits in these patients. This study will be the first study that will investigate whether cervical administration of ITB is safe in adults with SCI.

In addition to safety, efficacy will be exploratively assessed before phase 1 and at 3, 6, and 12 months after implantation of the ITB pump. The intermediate and long-term effects of cervical administration of ITB on respiratory muscles affecting pulmonary functions and SAS, as well as the effects of decreasing spasticity of the UE and LE on participants’ satisfaction, function, activities, and participation, will be assessed.

Besides the aforementioned direct effect of reduction of spasticity on UE function, reduction of spasticity may have an additional indirect benefit on UE function. At the end of conventional rehabilitation, UE reconstructive surgery is a well-documented and effective treatment in tetraplegia to improve arm or hand function [39]. Spasticity may adversely affect the outcome of this surgery and is mostly considered as a contraindication and must be treated first [40,41]. In case of generalized spasticity, cervical ITB may enable UE reconstructive surgery and thereby indirectly improve UE function.

When cervical ITB seems to be both safe and effective, it will change the usual care of this treatment. The catheter tip position should then be dependent on the localization of spasticity and treatment goals: tip at thoracolumbar level for functionally hindering spasticity of the LE muscles and tip at cervical level for functionally hindering spasticity of UE and LE muscles.

### Limitations

This study protocol has some limitations. Although the safety aim of the study is clear, it is important to note that this study is not powered to test for efficacy. It will provide us with the first exploratory data on efficacy, which can be used to power a subsequent study on efficacy. In addition, we anticipate variation in catheter placement relative to the lesion level. Although the catheter tip will deliberately be positioned just below the lesion to maximize sympathetic activity reduction, its proximity to the diaphragm may impact diaphragmatic function and, consequently, safety and efficacy. This variability will be considered when interpreting the results.

Although spasticity is a common complication in all central nervous system disorders with involvement of the upper motor neuron, only adults with SCI in a chronic phase will be included in this study. A potential follow-up study, in which adults with other disorders of the upper motor neuron and adults within 1 year after the onset of SCI can also be included, will focus more on effectiveness. However, the intensity of the current protocol may impact participants’ adherence and study feasibility, particularly in larger-scale or multicenter settings. Therefore, in this study, we will evaluate whether the frequency and scope of the postimplantation assessments can be reduced without compromising data quality or participant safety.

## Abbreviations

AHI: Apnea-Hypopnea Index
CE: Conformité Européenne
CPAP: continuous positive airway pressure
ITB: intrathecal baclofen
LE: lower extremity
MAS: Modified Ashworth Scale
MRC: Medical Research Council
MST: Medisch Spectrum Twente
ODI: Oxygen Desaturation Index
PGIC: Patient Global Impression of Change
PRPM: Perceived Resistance to Passive Movement
RCR: Roessingh Center for Rehabilitation
SAS: sleep apnea syndrome
SCI: spinal cord injury
SPIRIT: Standard Protocol Items: Recommendations for Interventional Trials
QIF-sf: Quadriplegia Index of Function–Short Form
UE: upper extremity
